# A Case Report on Dermatomyositis in a Female Patient with Facial Rash and Swelling

**DOI:** 10.21980/J8506D

**Published:** 2024-10-31

**Authors:** Rosalind Ma, Colin Danko

**Affiliations:** * University of Texas Southwestern Medical Center, Department of Emergency Medicine, Dallas, Tx

## Abstract

Early diagnosis of rheumatologic diseases can improve patient outcomes. While clinical presentations of rheumatologic diseases can be vague, dermatomyositis (DM) has distinctive cutaneous findings that can clue in providers towards the diagnosis. This is a case report of a 49-year-old female who presented with progressive facial swelling, rash, and generalized myalgias for a month. She had seen several outpatient providers and had one other emergency department (ED) visit for these symptoms prior to her diagnosis. She had already trialed steroid creams, antibiotics, and oral steroids with no significant improvement in her symptoms. A physical exam revealed peri-orbital edema, rash on her face, chest, and arms, and proximal muscle weakness. Lab work was significant for an elevated creatine kinase (CK). Rheumatology was consulted and recommended admission for expedited work-up for DM. The DM diagnosis was confirmed, and the patient was given intravenous immunoglobulin (IVIG) and discharged on oral steroids with dermatology and rheumatology outpatient follow-up. This case exemplifies how DM is often a missed diagnosis. However, by recognizing the classic dermatologic findings, conducting a muscle strength exam, and obtaining additional laboratory studies such as CK, the diagnosis can be made more easily.

**Topics:**

Dermatomyositis, weakness, rash, rheumatology, dermatology.

[Fig f1-jetem-9-4-v1] [Fig f2-jetem-9-4-v1] [Fig f3-jetem-9-4-v1] [Fig f4-jetem-9-4-v1] [Fig f5-jetem-9-4-v1]

**Figure f1-jetem-9-4-v1:**
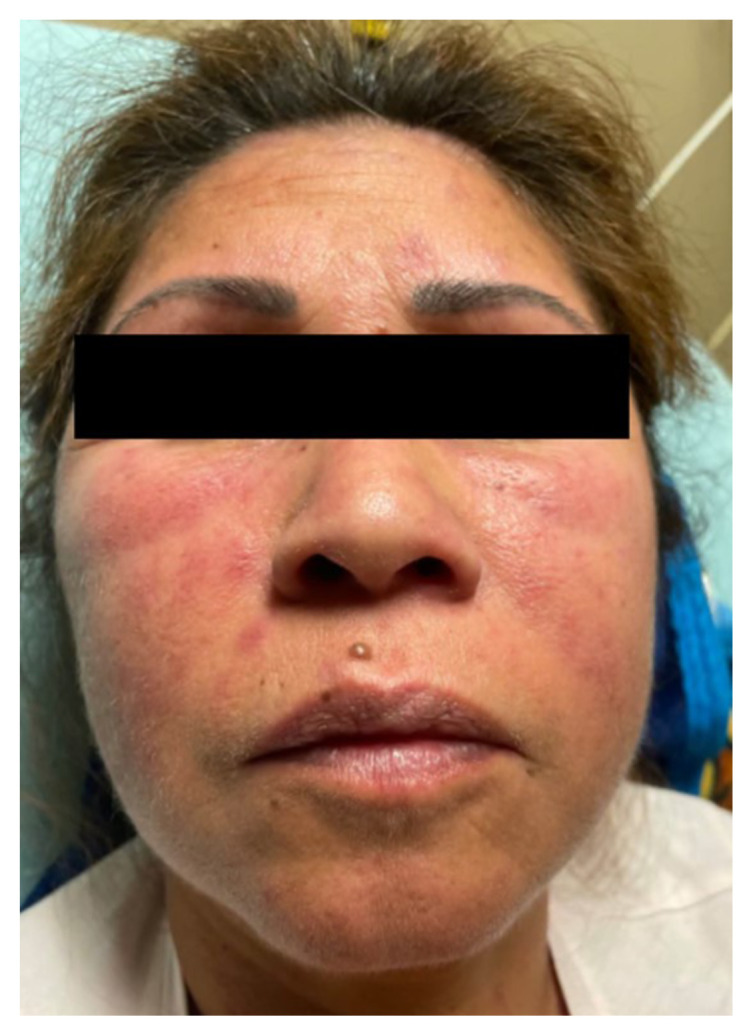


**Figure f2-jetem-9-4-v1:**
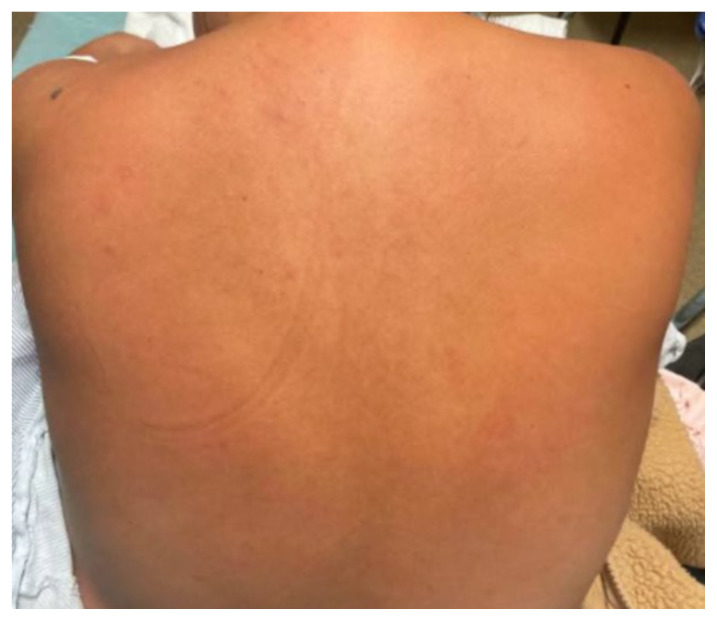


**Figure f3-jetem-9-4-v1:**
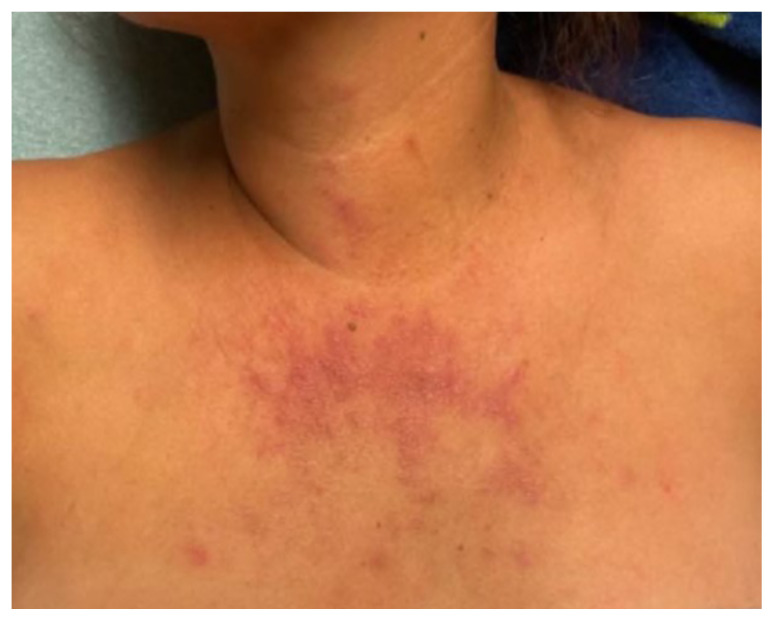


**Figure f4-jetem-9-4-v1:**
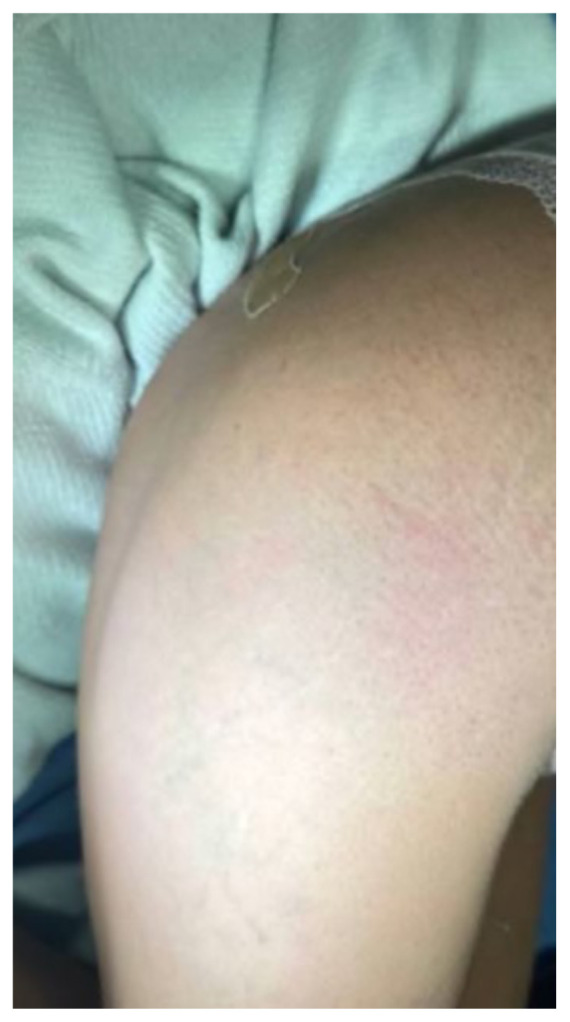


**Figure f5-jetem-9-4-v1:**
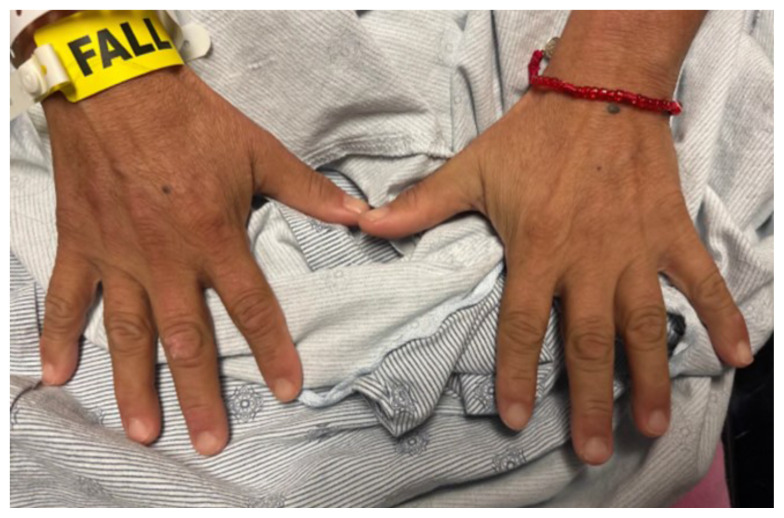


## Brief introduction

Dermatomyositis (DM) is an inflammatory myopathy that manifests with a classic purplish-red patchy rash and symmetrical proximal muscle weakness that can be complicated by respiratory failure. It is often associated with malignancies, often leading to poor patient outcomes.^1^, ^2^ DM is a rare condition with a prevalence of one in 100,000 in the general population.^3^ Early diagnosis of rheumatologic diseases has been demonstrated to improve morbidity and mortality in these patients.^4^, ^5^ While rheumatological emergencies are not common, patients with rheumatic complaints may encompass up to 8% of all emergency room visits, making it crucial for emergency medicine providers to keep these disease processes in their differentials.^6^, ^7^ Although the gold standard for diagnosing dermatomyositis is a muscle biopsy, there are characteristic cutaneous findings associated with DM that can aid in a potential clinical diagnosis as well. These dermatological findings include heliotrope rash, Gottron’s papules, and Shawl sign.^2^, ^8^

The presented case involves a patient who had several outpatient visits and two emergency department (ED) visits for progressive facial swelling, rash, and generalized weakness.

## Presenting concerns and clinical findings

A 49-year-old female with a past medical history of hypertension and diabetes presented to the ED on two separate occasions for facial swelling, rash, and generalized myalgias for the past month. Prior to her first ED visit, she had seen several other physicians for her symptoms and was given doxycycline and steroid creams without significant improvement. During her first ED visit, she reported generalized body aches, joint pain, and weakness. There was no known family history of autoimmune disease. Pictures of the rash were documented. Complete blood count (CBC), comprehensive metabolic panel (CMP), creatine kinase (CK), and inflammatory markers were obtained. These lab results revealed mildly elevated inflammatory markers and elevated CK. She was diagnosed with presumed contact dermatitis and discharged with continued oral steroids and a referral to dermatology. The patient returned to the ED two weeks later. It was prompted by persistent symptoms despite medication adherence. She reported the pruritic component of the rash had improved with oral steroids; however, the rash had expanded, and facial swelling had worsened. She additionally complained of progressive myalgias, newly developed proximal weakness, mild dysphagia, and dyspnea at rest. She denied new medications, irritants, or other exposures.

## Significant findings

The physical exam revealed significant periorbital swelling, facial edema, and a maculopapular rash across the upper chest, symmetrically across the extensor surfaces of the hands and the bilateral arms and thighs. The photograph of her face shows light-red to violaceous macules and patches, with inclusion of the nasolabial folds as well the forehead and upper eyelids with periorbital edema (heliotrope sign). The other rash images show “Shawl sign” (photograph of back showing erythema over the posterior aspect of the upper back), V sign (photograph of chest showing light-red violaceous plaque on mid-chest), Gottron’s papules (photograph of hands showing light red scaly papules overlying the right proximal interphalangeal joint [R PIP] and the metacarpophalangeal joint [MCP], and holster sign (photograph of thigh showing light red patches on bilateral lateral thighs). This distribution of rashes is pathognomonic for DM. In addition, the patient had proximal symmetric weakness in all four extremities. The remainder of her exam, including sensation, cranial nerves, and distal strength were unremarkable.

## Patient course

Given her second visit with worsening symptoms, repeat lab work (CBC, CMP, CK, and inflammatory markers) was obtained. The physical exam revealed a worsening rash compared to the prior on chart review and new proximal weakness. Lab work again showed an elevated CK. Given the distribution of the rash and proximal weakness, rheumatology was consulted and agreed with the concern for possible DM. Ultimately, they recommended admission for expedited work-up given the patient’s report of dysphagia and dyspnea. Dermatology was also consulted and agreed the presentation was consistent with DM. Knowing the association of this condition with malignancies, further lab work and imaging were obtained during her admission. No malignancies were identified. MRI of the lower extremities demonstrated myositis. Muscle biopsy was deferred but serology was positive for TIF-1y confirming DM diagnosis. The patient received two rounds of intravenous immunoglobulin (IVIG) and was continued on oral and topical steroids. She experienced significant symptomatic improvement with treatment and was ultimately discharged with outpatient clinic follow-up. Per review of follow-up outpatient visit documentation, the patient’s rash, myalgias, and weakness have all improved with immunotherapies.

## Discussion

Dermatomyositis is usually a clinical diagnosis, with patients often having the characteristic rash and elevated muscle enzymes. Some of the classic skin findings found include “Gottron’s sign” (symmetric skin changes over the extensor surfaces of the hands), heliotrope rash, edema around the upper eyelids, the “Shawl sign” (erythema over the posterior aspect of the neck or upper back that can extend to upper arms), “V sign” (diffuse rash across the upper chest), and “Holter sign” (rash on lateral thighs).

While most rashes are not emergent and are often deemed to be appropriate for outpatient workups, many low-income or uninsured patients are less likely to receive outpatient dermatologic care compared to privately insured patients.^9^ This forces many patients to rely on the ED for these complaints, resulting in skin complaints making up approximately 3% of ED visits.^10^ Even in this case, we can see how several providers had initially missed the diagnosis. Although cinching a dermatologic diagnosis can be difficult in the ED, we would like to suggest that DM can be more easily recognized because of two aspects. First, it has characteristic dermatologic findings and patterns which makes it more easily recognizable. Second, DM is uniquely associated with symmetric proximal muscle weakness and elevation in serum muscle enzymes such as CK and liver function tests. By performing a strength exam on these patients or asking focused questions on weakness and fatigue, providers can more easily diagnose DM earlier in the patient’s disease progression.

Dermatomyositis treatment often consists of outpatient therapies with steroids and immunosuppressants. However, sometimes patients develop dysphagia or respiratory insufficiency from their severe proximal weakness. In these cases, inpatient therapies may be pursued and often include more invasive therapies such as IVIG.^11^, ^12^ Additionally, DM can be associated with interstitial lung disease and malignancies.^13^ One study showed that there was at least a 10% mortality rate at the five years after diagnosis. The same study also showed that 16% of the patients who survived still had significant disability due to muscle weakness.^14^ These findings suggest that early diagnosis is associated with improved mortality and morbidity.^15^ While predominantly an outpatient diagnosis, we must recognize the ED is oftentimes the only initial access to healthcare for most uninsured patients. This makes it imperative that ED providers keep non-emergent autoimmune processes such as DM in their differential. If recognized by the ED provider, the patient can be referred appropriately to rheumatology and obtain earlier treatment, thus improving morbidity and mortality.

## Supplementary Information










