# Supplementary figures and images for: A Case Report on Dermatomyositis in a Female Patient with Facial Rash and Swelling
Source: J Educ Teach Emerg Med. 2024 Oct 31;9(4):V1–5. doi: 10.21980/J8506D (PMC11537729; doi:10.21980/J8506D)

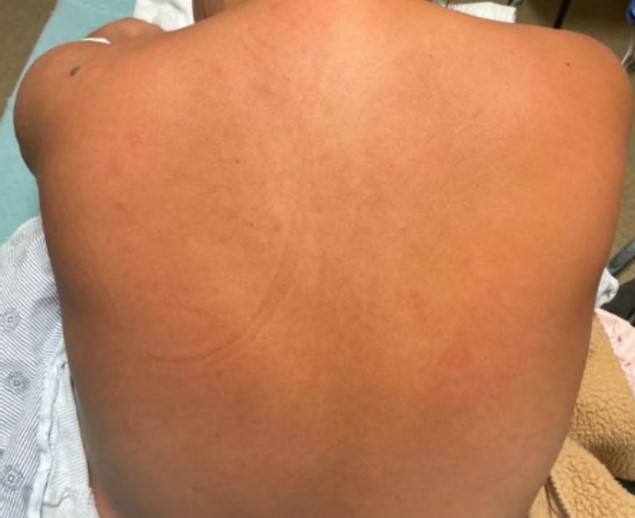

Supplement: Supplementary file 1 [file 9-4-V1-supp1.jpg]

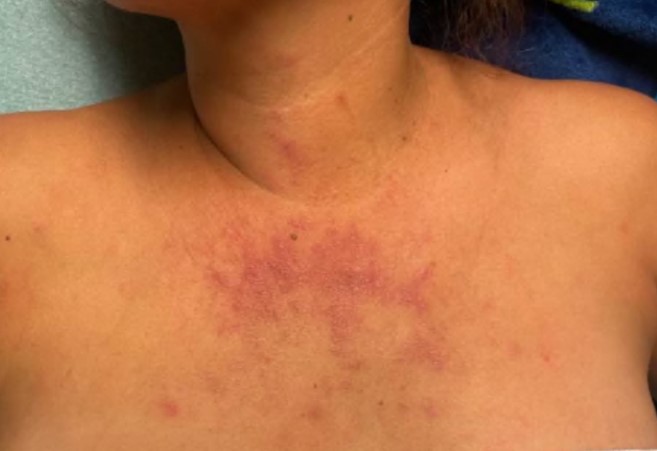

Supplement: Supplementary file 2 [file 9-4-V1-supp2.jpg]

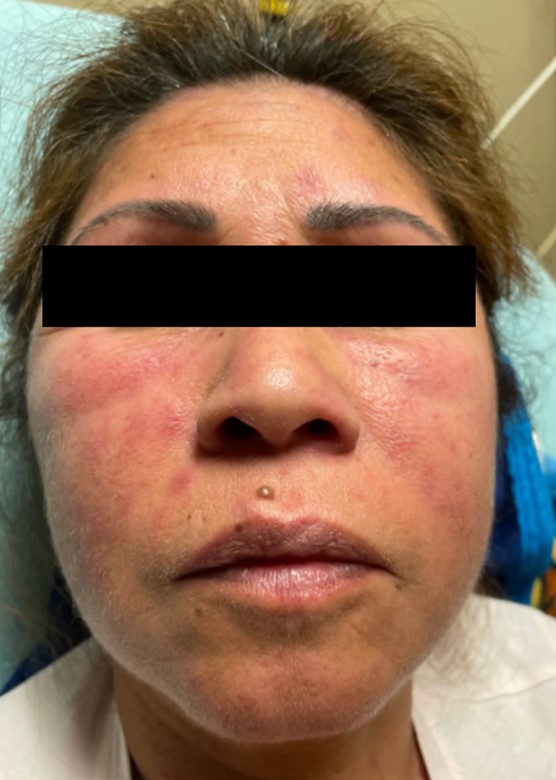

Supplement: Supplementary file 3 [file 9-4-V1-supp3.jpg]

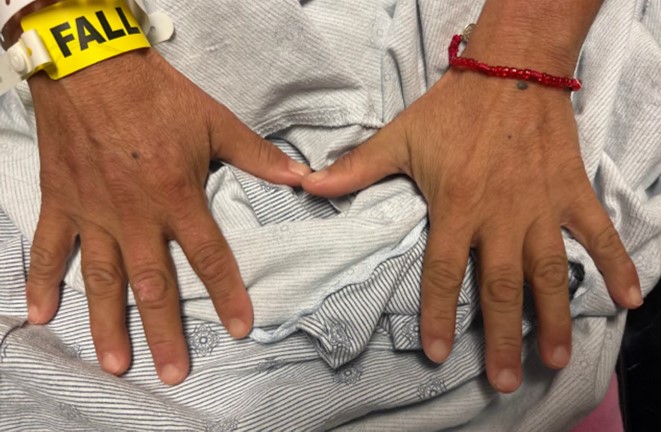

Supplement: Supplementary file 4 [file 9-4-V1-supp4.jpg]

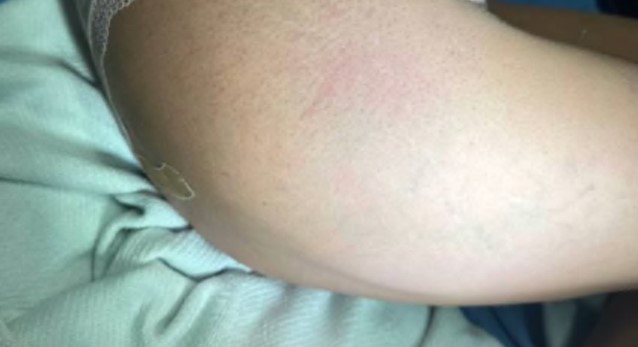

Supplement: Supplementary file 5 [file 9-4-V1-supp5.jpg]
